# *Abelmoschus manihot* Polysaccharides Target the AMPK/Nrf2/HO‐1 Signalling Pathway to Inhibit Macrophage M1 Polarisation and Hepatic Stellate Cell Activation Improving Liver Fibrosis in Mice

**DOI:** 10.1111/jcmm.71294

**Published:** 2026-07-26

**Authors:** Shixing Li, Zukuan Chang, Jinzhan Liu, Huilin Lu

**Affiliations:** ^1^ Department of Interventional Therapy Xinxiang Central Hospital Xinxiang Henan Province China

**Keywords:** *Abelmoschus manihot* polysaccharides, AMPK/Nrf2/HO‐1, hepatic stellate cells, liver fibrosis, macrophage polarisation

## Abstract

Liver fibrosis (LF) is a critical pathological stage in the progression from chronic liver disease to cirrhosis. While *Abelmoschus manihot* polysaccharides (AMP) demonstrate hepatoprotective potential, the underlying anti‐fibrotic mechanisms remain incompletely understood. A carbon tetrachloride‐induced LF mouse model was established followed by AMP intervention. Comprehensive assessments included ELISA measurement of serum biochemical indicators (ALT, AST, ALP, TBA, TBil), fibrosis markers (HA, PC III, LN, Hyp) and inflammatory cytokines (IL‐1β, TNF‐α, IL‐6, IL‐10). Liver tissue pathological changes were evaluated through haematoxylin–eosin (HE), Masson and Sirius Red staining. In vitro experiments utilised TGF‐β1‐stimulated JS‐1 and LPS/IFN‐γ‐induced M1‐type RAW264.7 macrophages. Experimental techniques encompassed MTT assay, immunofluorescence, qPCR, Western blot and flow cytometry to analyse cellular markers and signalling pathways. The study specifically investigated AMP's regulatory effects on macrophage polarisation and hepatic stellate cell activation, using the AMPK inhibitor (Compound C) to explore potential mechanistic pathways. AMP treatment effectively mitigated CCl_4_‐induced liver injury by reducing serum markers (ALT, AST, HA, PC III, Hyp) and pro‐inflammatory cytokines (IL‐1β, TNF‐α, IL‐6) while increasing anti‐inflammatory IL‐10. Mechanistically, AMP regulated macrophage polarisation (inhibiting M1 markers iNOS/CD80/CD86 while promoting M2 markers CD206/Arg‐1) and suppressed fibrogenesis by downregulating α‐SMA, Collagen I and TIMP1 while upregulating MMP13. These effects were mediated through activation of the AMPK/Nrf2/HO‐1 pathway, ultimately inhibiting HSCs activation and extracellular matrix (ECM) accumulation. The AMPK inhibitor Compound C attenuates these protective effects. AMP alleviates LF by activating the AMPK/Nrf2/HO‐1 pathway, modulating macrophage polarisation and ECM remodelling, demonstrating promising therapeutic potential.

**Abbreviations**
AMP:
*Abelmoschus manihot* polysaccharides
CLD: chronic liver disease
DAMP: damage‐associated molecular pattern
ECM: extracellular matrix
HSC: hepatic stellate cell
KC: Kupffer cell
LF: liver fibrosis
MAPK: mitogen‐activated protein kinase
MFBs: myofibroblasts
PILRα: paired immunoglobulin‐like Type 2 receptor alpha

## 1 Introduction

Liver fibrosis (LF) is a pivotal pathological process in chronic liver disease (CLD), marked by excessive extracellular matrix (ECM) accumulation resulting from activated hepatic stellate cells (HSCs), ultimately progressing to end‐stage liver disease [1]. If left untreated, LF may advance to cirrhosis, leading to hepatic dysfunction and potentially progressing to end‐stage complications including hepatic failure [2]. Research indicates that LF is a dynamic and reversible process that can be restored to near‐normal liver structure and function with appropriate intervention [3].

HSCs are the principal effector cells driving hepatic fibrogenesis. Under the influence of a liver injury microenvironment, HSCs transition from a dormant state into activated myofibroblasts (MFBs), acquiring pro‐inflammatory, proliferative, contractile and chemotactic properties. These activated cells then produce excessive ECM, promoting the progression of LF [4, 5]. As key immune regulators in LF progression, macrophages contribute to HSCs' apoptosis and ECM degradation. Macrophages undergo phenotypic transformation in response to diverse microenvironmental stimuli, predominantly differentiating into two primary subtypes [6]. M1 macrophages can release pro‐inflammatory factors (such as IL‐1β, TNF‐α, iNOS) during the initial stage of liver injury, exacerbating inflammatory responses, promoting HSCs' activation and accelerating LF [7]. Conversely, alternatively activated macrophages demonstrate the capacity to release immunomodulatory cytokines, including interleukin‐10 (IL‐10), transforming growth factor‐beta (TGF‐β) and arginase‐1 (Arg‐1), which exert anti‐inflammatory, tissue‐repair and immunoregulatory effects [8], while also degrading ECM and inhibiting LF [9]. The disruption of this polarisation balance may represent a critical link mechanism in LF progression.

In recent years, the role of the AMPK/Nrf2/HO‐1 signalling pathway in regulating macrophage polarisation has garnered significant attention. Activation of AMPK facilitates the polarisation of macrophages from the pro‐inflammatory M1 to the anti‐inflammatory M2 phenotype, thereby attenuating inflammation [10] and simultaneously upregulates HO‐1 expression by enhancing Nrf2 nuclear translocation [11]. HO‐1 serves as the rate‐limiting enzyme in heme catabolism, catalysing its breakdown into three biologically active products: bilirubin, carbon monoxide and ferrous iron. Endogenous CO exhibits potent immunomodulatory properties, suppressing LPS‐stimulated pro‐inflammatory cytokine production while upregulating anti‐inflammatory IL‐10 secretion in macrophages. Liu et al. discovered that Nrf2/HO‐1 pathway activation promotes hepatic antioxidant defences and attenuates fibrotic progression [12]. The Nrf2/HO‐1 signalling pathway activation demonstrates regulatory effects on macrophage polarisation, particularly inhibiting M1‐type activation while exhibiting anti‐inflammatory properties [13]. These discoveries highlight therapeutic targets for innovative fibrosis interventions.

*Abelmoschus manihot* polysaccharides (AMPs) are a class of naturally occurring high‐molecular‐weight compounds isolated and purified from the traditional medicinal plant *A. Manihot* [14]. These polysaccharides not only exhibit immunomodulatory activity but also demonstrate anti‐tumour effects against liver and gastric cancer [15, 16]. Despite AMP's therapeutic potential, its anti‐fibrotic mechanisms remain incompletely characterised. This investigation elucidates AMP's dual regulatory effects on HSC deactivation and macrophage phenotype switching through AMPK‐dependent Nrf2/HO‐1 pathway modulation, thereby advancing the mechanistic understanding of LF intervention.

## 2 Materials and Methods

### 2.1 Animal and Treatment

Fifty‐four male C57BL/6J mice (SPF, aged 5–6 weeks, body weight 18–20 g) were obtained from Spebio Biotechnology Co. Ltd. The animals were maintained in a temperature‐controlled facility (22°C–23°C) with a standardised 12 h light/12 h dark cycle and provided ad libitum access to food and water. A 1‐week acclimatisation period was implemented prior to experimental procedures to ensure environmental adaptation. After 1 week, 36 mice were randomly divided into six groups (*n* = 6): (1) Oil (HY‐108749, MedChemExpress) group; (2) CCl_4_ (HY‐Y0298, MedChemExpress) model group, in which mice received intraperitoneal injections of 15% CCl_4_/olive oil solution (2 mL/kg), administered 3 times per week for 6 weeks to establish a LF model. Starting from Week 3, CCl_4_‐treated mice were randomly divided into four groups: (3) Sora group (7.2 mg/kg, HY‐10201, MedChemExpress), a positive control [17]; (4) L‐AMP (100 mg/kg) group, with AMP preparation performed according to the method described by Wang et al. [16]; (5) M‐AMP (200 mg/kg) group; (6) H‐AMP (400 mg/kg) group. AMP and Sora treatment groups received respective drug solutions via intragastric administration for 3 weeks, while control and CCl_4_ groups received an equivalent volume of 0.1% carboxymethyl cellulose sodium (CMC‐Na, 9004‐32‐4, Solarbio) solution. At the 6‐week endpoint, all mice were humanely euthanised under isoflurane anaesthesia followed by collection of serum and hepatic tissue samples. Liver index = (wet liver weight/animal body weight) × 100%.

In order to deeply explore the mechanism of AMP in anti‐LF, 30 mice were randomly divided into five groups (*n* = 6): H‐AMP (400 mg/kg) group, Compound C (Com C, AMPK inhibitor, 10 mg/kg, B3252, APExBIO) group, H‐AMP + Com C group, H‐AMP + Com C + OE‐NC and H‐AMP + Com C + OE‐Nrf2 These five groups, together with the original Oil control group and CCl_4_ model group, constituted a total of seven experimental groups, with all experimental procedures performed according to the previously described methods. All animal experiments in this study were reviewed and approved by the Xinxiang Central Hospital Ethics Committee (Approval No.: 2024‐026‐01(K)). All the drug administration, tissue sampling, HE/Masson scoring and western blot/ELISA data reading were carried out by two independent experimental personnel without knowing the grouping information.

### 2.2 Serum Biochemical Indicators

Blood samples were incubated at 37°C for 1 h, followed by centrifugation (3000 rpm, 15 min, RT) to obtain serum, which was stored at −80°C. Serum ALT (MAK052, Sigma), AST (MAK055, Sigma), TBA (E003‐1‐1, Jiancheng), ALP (A059‐1‐1, Jiancheng), TBIL (BC5180, Solarbio) and HYP (BC0250, Solarbio) levels were measured using commercial kits according to the manufacturers' protocols. Prior to analysis, frozen serum was thawed at 4°C and mixed with the working solution. All assays were conducted using an automated biochemical analyser (HITACHI 7020, Hitachi).

### 2.3 Liver Histology

The liver specimens were fixed in 4% paraformaldehyde (PFA) at 4°C for 24 h before being processed through a graded ethanol series, embedded in paraffin and sectioned at 5 μm thickness. Histological evaluation was performed using three staining protocols: Haematoxylin and Eosin (HE, G1120, Solarbio), Masson's trichrome (G1340, Solarbio) and Sirius Red (G1473, Solarbio). For HE staining, tissue sections were heated at 65°C for 60 min, rehydrated through xylene and graded alcohol and stained with haematoxylin (5 min) followed by eosin counterstaining (30 s) with appropriate differentiation and washing steps. Masson staining involved sequential treatment with iron haematoxylin (7 min), Ponceau red (5 min), phosphomolybdic acid (2 min) and aniline blue (3 min). Sirius Red staining was performed by incubating sections with staining solution for 20 min in the dark. All stained sections were dehydrated through alcohol gradients, cleared in xylene and examined under a microscope (DM2500, Leica). Fibrosis assessment was conducted according to the established METAVIR scoring criteria [18], which evaluates both the degree and pattern of collagen deposition.

### 2.4 Cell Culture

The murine macrophage cell line RAW264.7 was provided by the Chinese Academy of Sciences Cell Bank (Shanghai, China), while JS‐1 cells (HSCs) were acquired from Fenghuibio (Changsha, China). Both cell lines were maintained in DMEM supplemented with 10% FBS (A5256701, Gibco) and 1% penicillin–streptomycin (SV30010, HyClone) at 37°C in a 5% CO_2_ atmosphere, with routine passaging every 48–72 h. In addition, an Nrf2‐overexpressing plasmid was constructed in this study and the transfection efficiency in RAW264.7 cells was verified by Western blotting.

### 2.5 Cell Treatment

To examine AMP's influence on JS‐1 cell activation, cells were allocated into five experimental groups: (1) Control group; (2) TGF‐β1 group treated with 5 ng/mL TGF‐β1 (GMP‐042, HuicH) for 48 h; (3) TGF‐β1 + 50 μg/mL AMP group; (4) TGF‐β1 + 100 μg/mL AMP group; (5) TGF‐β1 + 200 μg/mL AMP group. After co‐incubation with 5 ng/mL TGF‐β1 and specific AMP concentrations for 48 h, cells were used for subsequent experiments.

To assess AMP's impact on RAW264.7 polarisation, cells (1 × 10^6^/well) were plated in six‐well plates. When cell confluence reached approximately 70%, cells were divided into untreated and LPS/IFN‐γ treated groups and then further separated into five groups: (1) Control group; (2) LPS/IFNγ group, cells co‐treated with 100 ng/mL LPS (82857‐67‐8, Sigma‐Aldrich) and 2.5 ng/mL IFN‐γ (130‐105‐785, Miltenyi Biotec) for 24 h to induce M1 macrophage polarisation; (3) LPS/IFNγ + 50 μg/mL AMP; (4) LPS/IFNγ + 100 μg/mL AMP; (5) LPS/IFNγ + 200 μg/mL AMP, cells were subjected to combined treatment with LPS/IFN‐γ and varying concentrations of AMP for 24 h.

This study utilised a conditioned medium (CM) co‐culture system to examine macrophage‐mediated HSC activation. RAW264.7 macrophages were allocated into five treatment groups: (1) C‐CM group (control), with no treatment; (2) M1‐CM group, induced to M1 macrophage polarisation by stimulation with 100 ng/mL LPS and 2.5 ng/mL IFN‐γ for 12 h; (3) M1‐CM + AMP, pretreated with 100 μg/mL AMP for 24 h before M1 macrophage polarisation; (4) M1‐CM + Com C group, pretreated with 10 μM Com C for 24 h before M1 macrophage polarisation; (5) CM + AMP + Com C group, simultaneously pretreated with 100 μg/mL AMP and 10 μM Com C for 24 h before M1 macrophage polarisation. CM was prepared by collecting and filtering (0.22 μm) supernatants from each treatment group. JS‐1 cells were then plated at 1 × 10^6^ cells/well in six‐well plates. After adherence, the original medium was discarded and conditioned media from the differently treated RAW264.7 cells were added. Following 48 h of culture, cells were harvested for analysis.

### 2.6 MTT Assay

JS‐1 and RAW264.7 cells treated with different AMP concentrations were detached using 0.25% trypsin, collected and seeded at 1 × 10^4^ cells/well in 96‐well plates. Blank wells, a control group and treatment groups with varying AMP concentrations (0, 25, 50, 100, 200, 400, 800 μg/mL) were set up, with three replicates per group. Following 24 h culture, cells were treated with 50 μL serum‐free medium (51200038, Gibco) and 50 μL MTT solution (ab211091, Abcam), then incubated (37°C, 3 h). After adding 150 μL solubilisation buffer, plates were gently agitated (10 min) before measuring absorbance at 590 nm. Cell proliferation rates were calculated as follows: [(OD_experimental_ − OD_control_)/(OD_control_ − OD_blank_)] × 100%.

### 2.7 ELISA

Mouse serum was collected as described above. RAW264.7 cells were lysed in 500 μL ice‐cold PBS supplemented with protease inhibitors using ultrasonic disruption (SXSONIC, FS‐950N). Following centrifugation (12,000*g*, 20 min), supernatants were collected for analysis. Levels of IL‐1β, IL‐10, TNF‐α, IL‐6, HA, PC III, LN and iNOS in serum or cells were determined using commercial ELISA kits and measured with a microplate reader (MK4, Leica) according to the manufacturer's instructions.

Mouse IL‐1β (BMS6002‐2), IL‐10 (88‐7105‐22), TNF‐α (BMS607‐3TEN) and IL‐6 ELISA Kits (88‐7064‐22) were purchased from Invitrogen. HA (abx156663), PC III (abx154546), LN (abx154303) and iNOS kits (abx154467) were obtained from abbexa.

### 2.8 Immunohistochemistry and Immunofluorescence

Following deparaffinisation and rehydration, tissue sections underwent high‐pressure antigen retrieval in citrate buffer. After cooling to room temperature, slides were sequentially rinsed with tap water (5 min), treated with 3% H_2_O_2_ (10 min, RT) and washed with PBST. Sections were blocked with 1% bovine serum albumin (BSA, 9048‐46‐8, MedChemExpress) at room temperature for 1 h, then incubated overnight at 4°C with primary antibodies F4/80 (1:50, 123,101, Biolegend), α‐SMA (1:100, ab5694, Abcam) and Collagen 1 (1:100, ab278080, Abcam). After PBST washing, horseradish peroxidase (HRP)‐labelled secondary antibody (1:1000, ab6721, Abcam) was applied at room temperature for 1 h. Following PBST washing, sections were stained with diaminobenzidine (DAB, DA1010, Solarbio). After staining, the slides were gently washed with distilled water for 15 min, counterstained with haematoxylin (25 s) and rinsed under running tap water for an additional 15 min. Following dehydration, sections were neutral gum‐mounted, evaluated by light microscopy (Eclipse E100, Nikon) and stored at RT.

Tissue sections were deparaffinised, rehydrated, then subjected to heat‐mediated antigen retrieval. Cell slides underwent fixation (4% PFA, 20 min), PBS washes (3×) and permeabilisation (0.5% Triton X‐100, 10 min). Tissue sections and cell slides were blocked with 1% BSA at room temperature for 1 h, then incubated overnight at 4°C with primary antibodies CD68 (1:100, 28058‐1‐AP, Proteintech), iNOS (1:200, 13120T, Cell Signalling Technology), α‐SMA (1:100, 14395‐1‐AP, Proteintech) and Nrf2 (1:200, 12721T, Cell Signalling Technology). After washing 3 times with PBS, slides were incubated with FITC‐conjugated secondary antibodies (1:300, SA00003‐2, Proteintech) for 1 h. Following three PBS washes, nuclei were counterstained with DAPI (C1002, Beyotime) for 5 min and mounted with anti‐fade medium (P0126, Beyotime). Images were captured using a fluorescence microscope (CME20350C, Leica).

### 2.9 Western Blot

Mouse liver tissues or cells were collected and homogenised in RIPA buffer (89901, Thermo Scientific) containing 1% protease inhibitors, incubated on ice for 30 min and centrifuged at 12000 rpm for 30 min to collect the supernatant. Use the NE‐PER Nuclear and Cytoplasmic Protein Extraction Reagent (78833, Thermo) to extract nuclear and cytoplasmic proteins for detecting cytoplasmic and nuclear Nrf2 levels. Protein concentration was determined using a BCA protein assay kit (23227, Pierce). Equal amounts of protein were separated by SDS‐PAGE gel (5671033, Bio‐Rad) and transferred to a polyvinylidene fluoride (PVDF) membrane (1620174, Bio‐Rad). Membranes were blocked with 5% BSA at room temperature for 1 h, then incubated with primary antibodies overnight at 4°C. Primary antibodies were as follows. p‐AMPK (1:2000, ab133448), AMPK (1:2000, ab32047), keap‐1 (1:500, ab226997) and HO‐1 (1:2000, ab189491) from Abcam; α‐SMA (1:1000, bs‐10196R), MMP13 (1:500, bs‐0575R), TIMP1 (1:500, bsm‐62994R), CD80 (1:1000, bs‐1479R), CD86 (1:1000, bs‐43589R), CD206 (1:1000, bs‐23178R) and Collagen 1 (1:500, bs10423R) from Bioss; Fibronectin (1:1000, 30903T), iNOS (1:1000, 13120T), Arg‐1 (1:1000, 93668T), Nrf2 (1:200, 12721T) from Cell Signalling Technology; GAPDH (1:5000, 10494‐1‐AP) and TGF‐β1 (1:1000, 21898‐1‐AP) from Proteintech. The membranes were washed with PBST, followed by incubation with a secondary antibody (1:2000, ab6932, Abcam) at room temperature for 1 h. Detection was performed using an ECL chemiluminescence detection system (32209, Thermo Scientific) and the protein bands were visualised and quantified using Gel‐Pro Analyser 3.0 software.

### 2.10 qPCR

Total RNA was isolated from hepatic tissues or cultured cells using TRIzol reagent (15596018CN, Invitrogen), followed by cDNA synthesis using a reverse transcription kit (RR047A, TAKARA). The qPCR reaction system contained 2.5 μL cDNA template, 1 μL gene‐specific primers and TB Green Fast qPCR Mix (RR430A, TAKARA) in a final volume of 20 μL. Thermal cycling parameters included: initial denaturation at 95°C for 5 min; 40 cycles of denaturation at 95°C for 10 s and annealing/extension at 60°C for 1 min. Gene expression was normalised to GAPDH as the housekeeping gene, with relative quantification calculated using the 2^−ΔΔ*C*t^ algorithm. Corresponding primer sequences are detailed in Table 1.

**TABLE 1:** Primer sequences for qPCR‐related genes.

Gene	Forward primer	Reverse primer
GAPDH	GGCTATCACGGAGGCTGTGAA	CCAGCCTTAGCATCAAAGATGGA
CD80	CTTTCAGACCGGGGCACATA	GAAGCGAGGCTTTGGGAAAC
CD86	TCAAGACATGTGCAACCTGC	CCTTTGTAAATGGGCACGGC
CD206	GCTTCCGTCACCCTGTATGC	TCATCCGTGGTTCCATAGACC
Arg‐1	AGCTCTGGGAATCTGCATGG	ATGTACACGATGTCTTTGGCAGATA
iNOS	ATTCACAGCTCATCCGGTACG	GGATCTTGACCATCAGCTTGC

### 2.11 Flow Cytometry

Murine macrophage RAW264.7 cells were collected and centrifuged at 1500 rpm for 5 min at 4°C. Following initial separation, cells were subjected to triple phosphate‐buffered saline washing procedures using pre‐chilled solution. Subsequently, cellular specimens were carefully resuspended in standardised binding buffer, achieving a precise cellular concentration of 1 × 10^6^ cells per millilitre. Cell suspensions (100 μL) were stained with CD80‐APC (A14724, Invitrogen) and CD86‐FITC (A18658, Invitrogen) antibodies for 20 min at room temperature, protected from light. Following three PBS washes, cells were resuspended in 400 μL 1 × Binding buffer and analysed on a flow cytometer (Beckman, USA). A minimum of 1 × 10^5^ events were acquired and analysed with FlowJo software.

### 2.12 Statistical Analysis

Statistical analysis was conducted utilising SPSS version 20.0 and GraphPad Prism 8.0.2 computational platforms. Quantitative results were presented as arithmetic mean with standard deviation (mean ± SD). First, a Shapiro–Wilk normality test is conducted for each group of data. For normally distributed data with homogeneous variances, the inter‐group differences are analysed using a two‐sided independent sample *t*‐test (for two groups) or one‐way analysis of variance (ANOVA), followed by post hoc analysis using Tukey's multiple comparison test. When the data do not meet the normality assumption, non‐parametric tests are employed: two‐group comparisons use the Mann–Whitney *U* test and multi‐group comparisons use the Kruskal–Wallis *H* test. After significance, further multiple comparison tests using Dunn–Bonferroni are conducted. The threshold for statistical significance was predetermined at *p* < 0.05.

## 3 Result

### 3.1 AMP Alleviates CCl_4_‐Induced Liver Cell Injury and Inflammation

**FIGURE 1:**
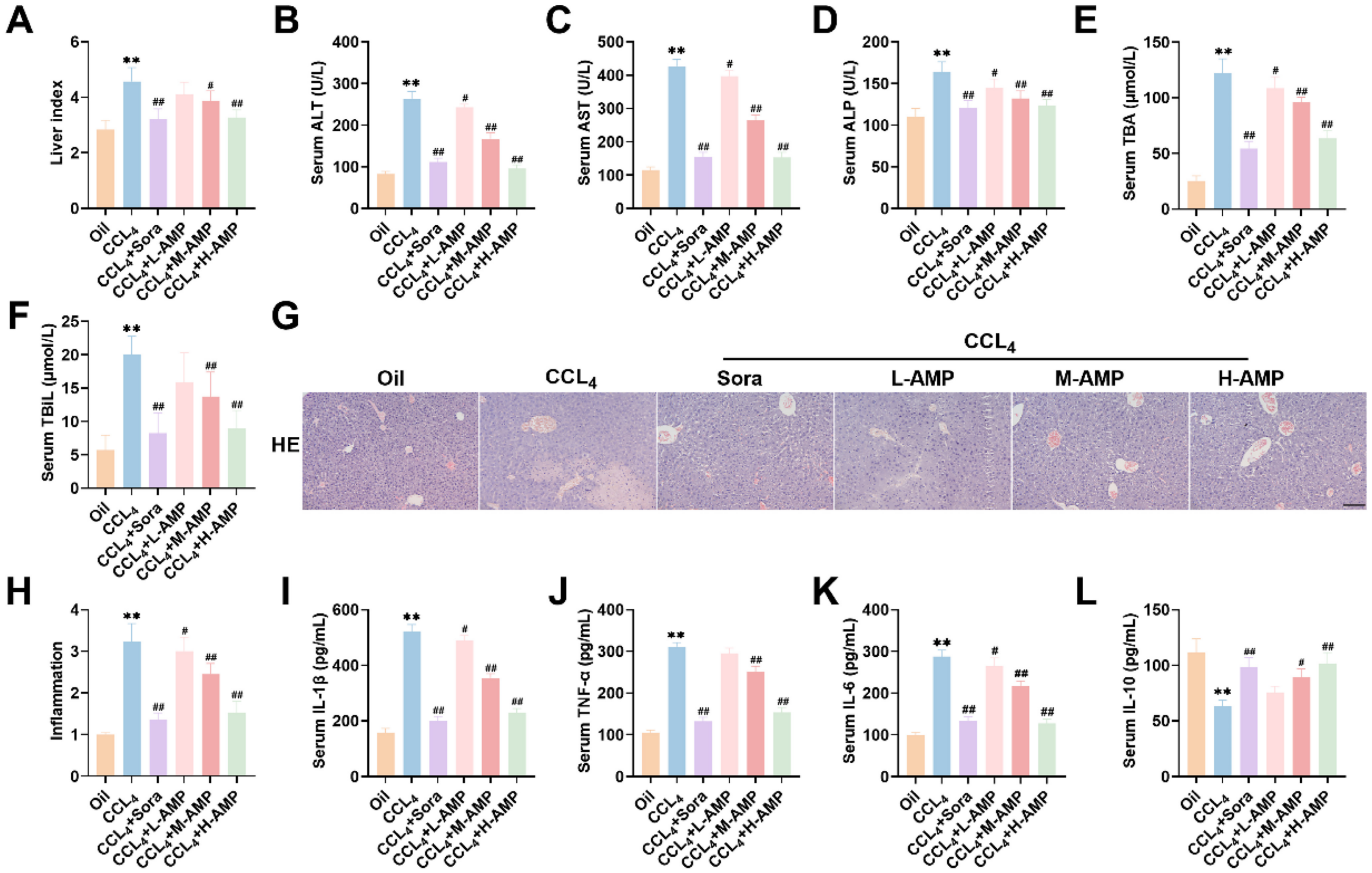
AMP Alleviates CCl_4_‐induced mouse hepatocellular injury and inflammation. (A) Mice were randomised into six groups: Oil group and CCl_4_ model group. LF was induced via 6‐week thrice‐weekly 15% CCl_4_/olive oil i.p. injections. From Week 3, CCl_4_‐treated mice were randomised into four intervention groups: Sora and AMP (100, 200, 400 mg/kg). Liver injury was assessed by liver coefficient. (B–F) Quantitative analysis of serum liver function damage indicators (ALT, AST, ALP) and bile acid metabolism markers (TBA, TBiL) to investigate AMP's effects on CCl_4_‐induced mouse hepatocellular injury and cholestasis. (G, H) HE staining analysis of liver tissue pathological changes in each group, evaluating AMP's impact on CCl_4_‐induced mouse liver tissue. Scale bar 100 μm. (I–L) ELISA detection of pro‐inflammatory cytokines (IL‐1β, TNF‐α, IL‐6) and anti‐inflammatory factor (IL‐10) levels in mouse serum to assess AMP's effect on CCl_4_‐induced inflammation. Data are presented as mean ± SD from 6 biologically independent animals (*n* = 6). **p < 0.01 versus oil; ^#^ *p <* 0.05, ^##^ *p <* 0.01 versus CCl_4_.

To assess the impact of AMP on CCl_4_‐triggered hepatocyte damage and inflammatory responses, key biochemical and histopathological markers of liver injury and inflammation were evaluated. During LF, liver tissue undergoes structural changes accompanied by inflammation, cell infiltration or regenerative nodules, which can lead to liver enlargement and increased weight. The liver index serves as an indirect indicator of fibrosis severity. The CCl_4_ model group exhibited a markedly higher liver index compared to the Oil group. However, AMP administration (L‐AMP, M‐AMP, H‐AMP) dose‐dependently attenuated this elevation, with H‐AMP demonstrating the most pronounced inhibitory effect—similar to that observed in the Sora positive control group (Figure 1A). Serum biochemical evaluation showed significantly increased concentrations of hepatocellular injury markers (ALT, AST, ALP) and cholestatic parameters (TBA, TBil) in CCl_4_‐challenged mice compared to vehicle control animals, validating the successful development of CCl_4_‐induced hepatocyte injury with associated biliary stasis. After AMP intervention, these biochemical indicators showed dose‐dependent reduction, with the high‐dose AMP group demonstrating therapeutic effects comparable to the positive control Sora group, indicating the potent hepatoprotective activity of AMP (Figure 1B–F). These results indicate that AMP effectively attenuates CCl_4_‐induced mouse liver cell injury, alleviates bile acid stasis associated with LF and that 400 mg/kg AMP achieves efficacy similar to Sora.

Histopathological examination by HE staining revealed extensive hepatic damage in the CCl_4_ model group, characterised by pronounced lipid vacuolation, marked inflammatory infiltration, disrupted hepatic architecture and evident hepatocellular necrosis. AMP treatment could dose‐dependently improve liver tissue structure, characterised by reduced inflammatory cell infiltration, decreased necrotic areas and restoration of hepatocyte morphology. The H‐AMP group demonstrated the most significant improvement in liver pathology, approaching the Sora group (Figure 1G). According to the METAVIR scoring system, fibrosis severity was quantitatively assessed, revealing that the CCl_4_ model group exhibited severe LF, while AMP treatment significantly attenuated fibrosis progression (Figure 1H). ELISA quantification revealed elevated serum concentrations of pro‐inflammatory mediators (IL‐1β, TNF‐α, IL‐6) concomitant with reduced IL‐10 levels in CCl_4_‐administered mice, exacerbating liver inflammation and promoting fibrosis progression and hepatocyte injury. AMP and Sora treatments significantly suppressed pro‐inflammatory mediators while enhancing anti‐inflammatory factors, with AMP demonstrating dose‐dependent anti‐inflammatory efficacy (Figure 1I–L), indicating its therapeutic potential for mitigating hepatic inflammation and cellular damage in the experimental model.

### 3.2 AMP Improves CCl_4_‐Induced LF in Mice

**FIGURE 2:**
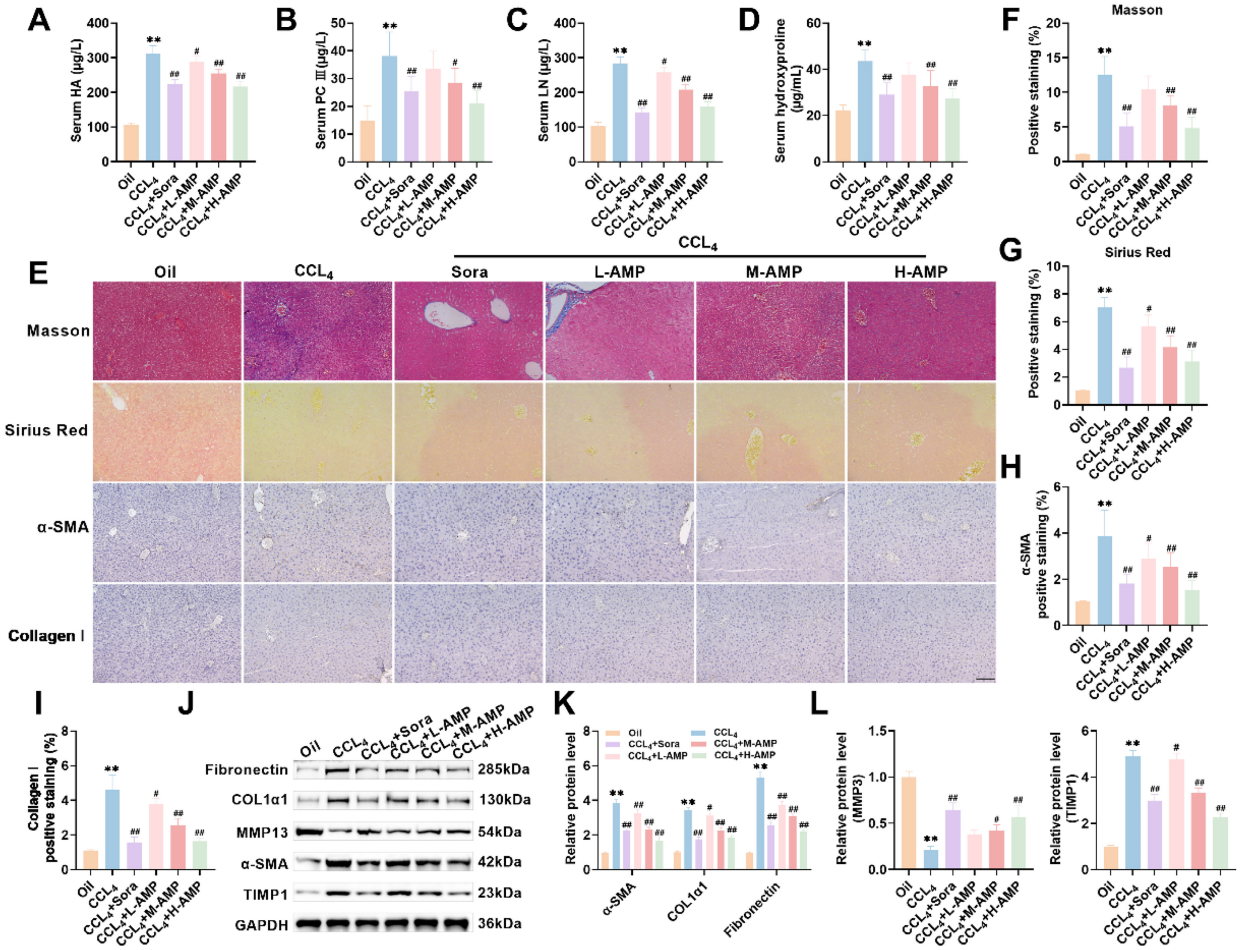
AMP improves CCl_4_‐induced LF. (A–D) ELISA detection of liver injury serological markers (HA, PC III, LN, Hyp) in each group to evaluate AMP's effects on CCl_4_‐induced LF. (E) Liver tissue from each group underwent Masson, Sirius Red staining and immunohistochemistry (α‐SMA, Collagen I) to analyse the impact of different AMP concentrations on HSCs activation, ECM deposition and LF progression through pathological histological analysis. Scale bar 100 μm. (F, G) Positive percentage (%) of Masson and Sirius Red staining. (H, I) Positive percentage (%) of α‐SMA and Collagen I. (J–L) Western blot was performed to detect LF‐related protein expression levels (α‐SMA, Collagen I, Fibronectin, MMP13 and TIMP1) to assess AMP's effects on the molecular mechanism of CCl_4_‐induced LF. Data are presented as mean ± SD from six biologically independent animals (*n* = 6). **p < 0.01 versus oil; ^#^ *p* < 0.05, ^##^ *p* < 0.01 versus CCl_4_.

Through the development of a carbon tetrachloride‐induced LF murine model, the current investigation systematically evaluated both the therapeutic efficacy and underlying molecular pathways of AMP in liver fibrogenesis. In LF research, serum markers (HA, PC III, LN) and Hyp serve as critical indicators reflecting ECM metabolic balance and are used to assess LF severity [19]. Compared to the control group, serum levels of HA, PC III, LN and Hyp were significantly elevated in the CCl_4_ mouse model, indicating enhanced ECM deposition and disrupted metabolic balance. After AMP and Sora treatment, these markers showed varying degrees of reduction, with AMP exhibiting a notable dose‐dependent effect. The high‐dose AMP intervention produced the most significant improvement, comparable to the positive control (Figure 2A–D). Collagen fibre deposition represents a hallmark pathological feature of ECM accumulation following HSC activation and is a key histological characteristic of mouse LF progression. Histopathological analysis using Masson's trichrome and Sirius Red staining demonstrated substantial collagen accumulation in hepatic tissues following CCl_4_ exposure. Importantly, AMP administration exhibited a concentration‐dependent attenuation of ECM deposition. The H‐AMP group showed the most remarkable effect, comparable to the positive control Sora treatment (Figure 2E–G), highlighting AMP's potential to effectively interrupt critical fibrosis progression pathways.

At the molecular level of progressive hepatic fibrogenesis, Fibronectin serves as an early fibrosis‐sensitive marker that promotes HSCs activation. Activated HSCs demonstrate characteristic α‐SMA expression and robust Collagen I production, which drives pathological ECM accumulation. MMP13, as a key collagen‐degrading enzyme, maintains tissue homeostasis through specific ECM hydrolysis, while TIMP1 selectively inhibits its activity. The dynamic balance between these factors jointly regulates ECM metabolism. Immunohistochemical analysis revealed marked upregulation of α‐SMA and Collagen I expression in hepatic tissues from CCl_4_‐treated mice (Figure 2E,H,I). Western blot results demonstrated that CCl₄ exposure markedly elevated hepatic fibrogenic markers (α‐SMA, Collagen I, Fibronectin and TIMP1) while suppressing MMP13 expression, validating the successful induction of experimental LF. AMP treatment reverses the alterations of the aforementioned fibrosis marker proteins in a dose‐dependent manner and the effect of the high‐dose AMP is comparable to that of the Sore group (Figure 2J–L). In summary, AMP can curb the heightened ECM degradation capacity, diminish collagen‐fibre deposition and ameliorate CCl_4_‐induced hepatic fibrosis in mice.

### 3.3 AMP Reduces Macrophage Infiltration and Modulates Macrophage Polarisation in CCl_4_‐Induced LF in Mice

**FIGURE 3:**
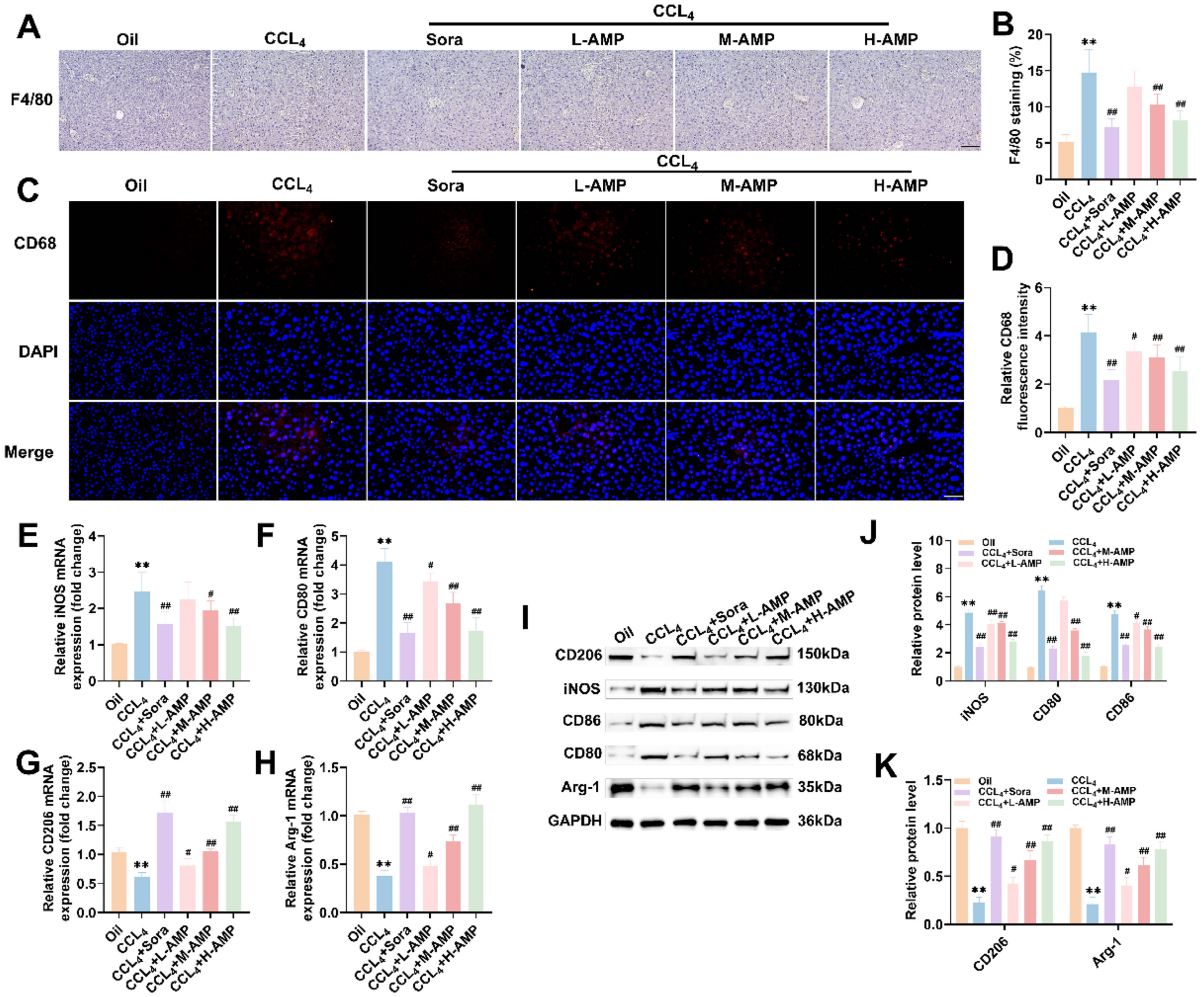
AMP reduces macrophage infiltration and modulates macrophage polarisation in CCl_4_‐induced LF. (A, B) Immunohistochemical detection of F4/80 expression levels in liver tissues to evaluate AMP's impact on macrophage infiltration. Scale bar 100 μm. (C, D) Immunofluorescence detection of CD68 (macrophage marker) expression levels in liver tissues from each group to assess AMP's effect on macrophage infiltration. Scale bar 100 μm. (E–H) qPCR analysis of macrophage polarisation marker expression levels (M1: CD80, CD86; M2: CD206, Arg‐1) in liver tissues from each group to evaluate AMP's influence on macrophage polarisation in CCl_4_‐induced mouse liver tissues. (I–K) Western blot further analysis of macrophage polarisation marker expression levels (M1: iNOS, CD80, CD86; M2: CD206, Arg‐1) in liver tissues from each group to assess AMP's effect on macrophage polarisation in CCl_4_‐induced mouse liver tissues. Data are presented as mean ± SD from 6 biologically independent animals (*n* = 6). **p < 0.01 versus oil; ^#^ *p* < 0.05, ^##^ *p* < 0.01 versus CCl_4_.

This study investigated whether AMP attenuates CCl_4_‐induced LF by regulating macrophage infiltration and polarisation through dynamic assessment of macrophage accumulation and polarisation marker expression. Histochemical and fluorescence imaging demonstrated increased expression of macrophage markers F4/80 and CD68 in CCl_4_‐exposed hepatic tissues, suggesting macrophage involvement in fibrogenesis. Notably, AMP treatment dose‐dependently inhibited F4/80 and CD68 expression levels, with the high‐dose AMP intervention showing effects comparable to the positive control (Figure 3A–D), indicating that AMP effectively suppresses pathological macrophage infiltration.

Molecular analyses demonstrated a marked shift towards M1 polarisation in fibrotic livers, evidenced by elevated expression of iNOS, CD80 and CD86, coupled with reduced CD206 and Arg‐1 levels compared to controls. These findings indicate the predominance of pro‐inflammatory M1 macrophages during hepatic fibrogenesis. After AMP treatment, the expression of iNOS, CD80 and CD86 in liver tissues of CCl_4_‐induced mice significantly decreased, while CD206 and Arg‐1 expression markedly increased, showing a dose‐dependent manner (Figure 3E–K). These findings indicate that AMP exerts dual therapeutic effects by limiting macrophage infiltration and promoting a phenotypic shift from M1 to anti‐inflammatory M2 macrophages, thereby attenuating hepatocellular injury, inflammatory responses and CCl_4_‐induced fibrogenesis.

### 3.4 AMP Activates AMPK/Nrf2/HO‐1 Signalling Pathway to Inhibit Macrophage M1 Polarisation and HSC Activation, Alleviating CCl_4_‐Induced LF in Mice

**FIGURE 4:**
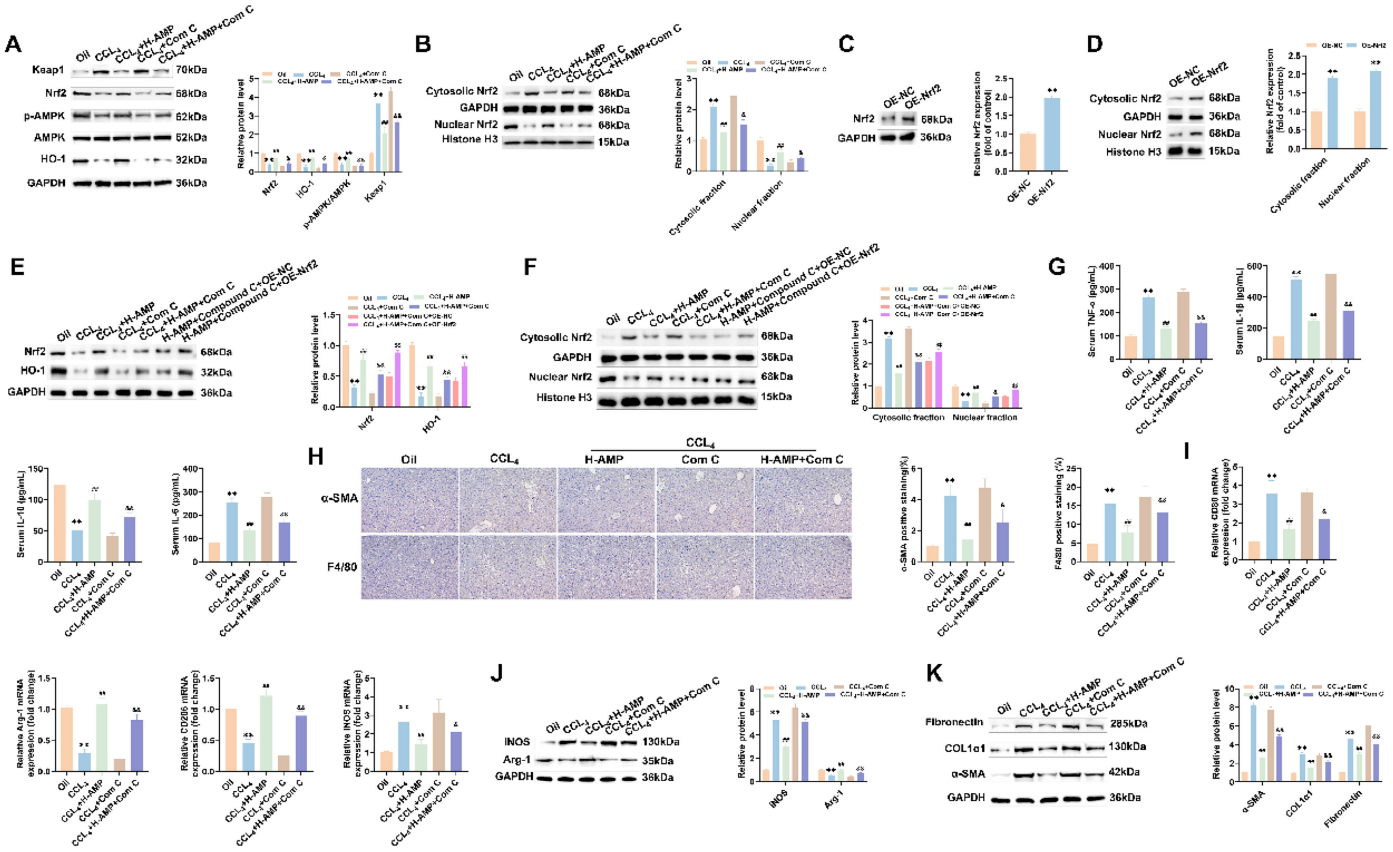
AMP activates AMPK/Nrf2/HO‐1 signalling pathway to inhibit macrophage M1 polarisation and hepatic stellate cell activation, alleviating CCl_4_‐induced LF. (A) Intervention with high‐dose AMP (400 mg/kg), AMPK inhibitor (Com C) and their combination in a CCl_4_‐induced LF mouse model, using Western blot to analyse expression changes of AMPK/Nrf2/HO‐1 axis‐related proteins (p‐AMPK, AMPK, Nrf2, Keap1, HO‐1) in liver tissues from each group, evaluating AMP's impact on the AMPK/Nrf2/HO‐1 signalling pathway. (B) To verify the effect of Nrf2 overexpression, the levels of Nrf2 in the cytoplasm and nucleus were respectively detected. (C, D) Western blot assay was used to investigate the effects of expressing Nrf2 on the Nrf2 protein, cytoplasm and nucleus in the liver tissues of mice. (E) Western blot analysis of total Nrf2 and HO‐1 protein expression. (F) Subcellular fractionation showing cytoplasmic and nuclear Nrf2 levels. (G) Liver tissue levels of pro‐inflammatory cytokines (TNF‐α, IL‐6, IL‐1β) and anti‐inflammatory IL‐10 were quantified by ELISA to evaluate AMP's anti‐inflammatory effects. (H) Immunohistochemical analysis of macrophage marker (F4/80) and HSCs activation marker (α‐SMA) expression levels in each group, evaluating AMP's impact on macrophage infiltration and HSCs activation. Scale bar 100 μm. (I) qPCR detection of M1 macrophage (iNOS, CD80) and M2 macrophage polarisation markers (Arg‐1, CD206) to assess AMP's influence on macrophage polarisation. (J) Western blot further analysing macrophage polarisation markers (iNOS, CD206) in mouse liver tissues to explore AMP's effect on macrophage polarisation through targeting the AMPK/Nrf2/HO‐1 signalling pathway. (K) Western blotting was performed to quantify HSCs activation markers (α‐SMA, Collagen I, Fibronectin), evaluating AMP's inhibitory effects via the AMPK/Nrf2/HO‐1 pathway. Data are presented as mean ± SD from six biologically independent animals (*n* = 6). **p < 0.01 versus oil; ^##^ *p* < 0.01 versus CCl_4_; ^&^ *p* < 0.05, ^&&^ *p* < 0.01 versus H‐AMP.

Exploring the fundamental signalling mechanisms enabling AMP's protective effects against hepatic fibrotic transformation through AMPK/Nrf2/HO‐1 pathways. We intervened in the CCl_4_‐induced LF mouse model using high‐dose AMP (400 mg/kg), Com C (AMPK inhibitor) and their combination. Western blot, ELISA, immunohistochemistry and qPCR were employed to systematically analyse the regulatory role of this pathway in LF progression. Western blot analysis revealed that CCl_4_ treatment significantly downregulated Nrf2, HO‐1 and p‐AMPK/AMPK expression while upregulating Keap1, indicating suppression of the AMPK/Nrf2/HO‐1 pathway. In contrast, AMP treatment markedly increased Nrf2, HO‐1 and p‐AMPK/AMPK levels while reducing Keap1, suggesting AMP activates this pathway. Conversely, Com C exerted opposing effects, further inhibiting the pathway. Co‐treatment with AMP and Com C markedly reduced hepatic Nrf2, HO‐1 and p‐AMPK/AMPK levels while elevating Keap1 expression versus AMP monotherapy, verifying Com C's inhibitory effect on AMP‐mediated AMPK/Nrf2/HO‐1 pathway activation (Figure 4A). The further nuclear‐cytoplasmic separation Western blot results showed that CCL4 treatment decreased the nuclear level of Nrf2 and increased the cytoplasmic level of Nrf2; AMP treatment promoted the nuclear translocation of Nrf2, while Com C could inhibit this translocation process (Figure 4B). To further explore the AMPK/NRF2/HO‐1 signalling pathway, this study constructed a plasmid overexpressing Nrf2 and confirmed that it could effectively upregulate the protein expression of Nrf2 in mouse liver tissues (Figure 4C,D). Overexpression of Nrf2 on the basis of AMP + Com C could significantly upregulate the protein expression of Nrf2 and HO‐1 as well as restore the nuclear level of Nrf2 (Figure 4E,F). ELISA results further demonstrated that co‐treatment with AMP and Com C suppressed AMP's anti‐inflammatory effects (Figure 4G). The results demonstrate that AMP ameliorates CCl_4_‐triggered hepatic fibrosis through AMPK/Nrf2/HO‐1 pathway activation, leading to attenuated inflammatory responses and reduced fibrotic progression.

Immunohistochemistry, qPCR and Western blot analyses further demonstrated that co‐treatment with AMP and Com C, compared with AMP alone, significantly attenuated AMP's ability to suppress M1 polarisation and to promote M2 polarisation of macrophages in mouse liver tissue (Figure 4H–J). Additionally, immunohistochemistry and Western blot demonstrated that AMP markedly decreased the expression of α‐SMA, Collagen I and Fibronectin in liver tissues compared to the CCl_4_ model, whereas Com C alone had no significant effect (Figure 4H,K). Furthermore, combined treatment with AMP and Com C significantly reversed the inhibitory effect of AMP. These results confirm that AMP alleviates CCl_4_‐induced LF by activating the AMPK/Nrf2/HO‐1 pathway, thereby inhibiting macrophage infiltration and M1 polarisation, promoting M2 polarisation, mitigating inflammation, suppressing HSCs activation and reducing ECM deposition.

### 3.5 AMP Inhibits HSCs Activation In Vitro

**FIGURE 5:**
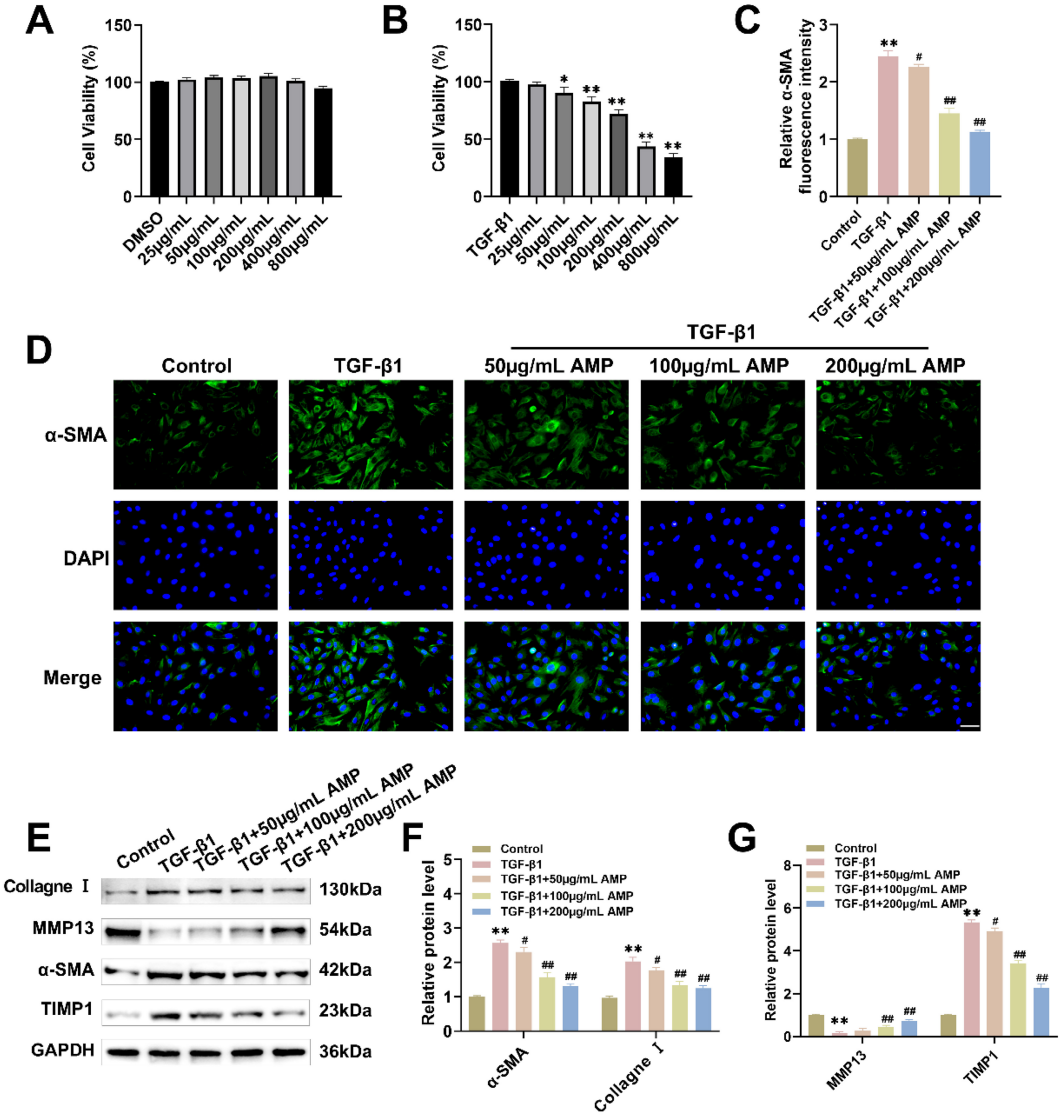
AMP inhibits HSCs activation in vitro. (A) MTT assay detecting the viability of JS‐1 cells at different AMP concentrations. (B) MTT assay detecting the viability of JS‐1 cells treated with TGF‐β1 at different AMP concentrations. *p < 0.05, **p < 0.01 versus TGF‐β1. (C, D) Immunofluorescence detection of α‐SMA expression (HSCs activation marker) in JS‐1 cells from each group to evaluate the effect of different AMP concentrations on HSCs activation in vitro. Scale bar 50 μm. (E–G) To evaluate the in vitro effects of different AMP concentrations on LF, Western blot analysis was performed to detect the expression levels of fibrosis‐related proteins (α‐SMA, Collagen I, MMP13 and TIMP1) in JS‐1 cells. Data are presented as mean ± SD from three biologically independent experiments (*n* = 3). **p < 0.01 vs. Control; # *p* < 0.05, # *p* < 0.05, ## *p* < 0.01 vs. TGF‐β1.

The MTT assay demonstrated concentration‐dependent effects of AMP on JS‐1 cells, with lower concentrations (25, 50, 100, 200 μg/mL) showing modest proliferative enhancement, while higher doses (400, 800 μg/mL) exhibited inhibitory effects on cell growth, though all tested concentrations maintained cell viability within normal ranges (Figure 5A). TGF‐β1 is widely recognised as a pivotal mediator of hepatic fibrogenesis, potently inducing HSCs transdifferentiation into collagen‐producing MFBs and driving excessive ECM accumulation [20]. In the TGF‐β1‐induced JS‐1 cells LF model, AMP exhibited cell viability inhibition, particularly at concentrations of 400 and 800 μg/mL, which significantly reduced JS‐1 cell viability (Figure 5B). Based on dose–response characteristics and cellular viability assessments, 50, 100 and 200 μg/mL AMP was selected for further investigation. Immunofluorescence analysis revealed TGF‐β1‐stimulated JS‐1 cells exhibited markedly elevated α‐SMA expression relative to untreated controls. AMP treatment dose‐dependently inhibited α‐SMA expression, effectively suppressing HSCs activation (Figure 5C,D). Western blot further verified this finding. TGF‐β1‐treated JS‐1 cells significantly increased protein levels of α‐SMA, Collagen 1 and TIMP 1, while decreasing MMP13 protein levels, reflecting the typical LF cell phenotype. Notably, AMP administration effectively suppressed the expression of key fibrotic markers while demonstrating dose‐dependent enhancement of MMP13, a critical matrix‐degrading protease (Figure 5E–G). These in vitro experiments demonstrate that AMP can inhibit HSCs proliferation and activation, thereby improving LF.

### 3.6 AMP Inhibits Macrophage M1 Polarisation In Vitro

**FIGURE 6:**
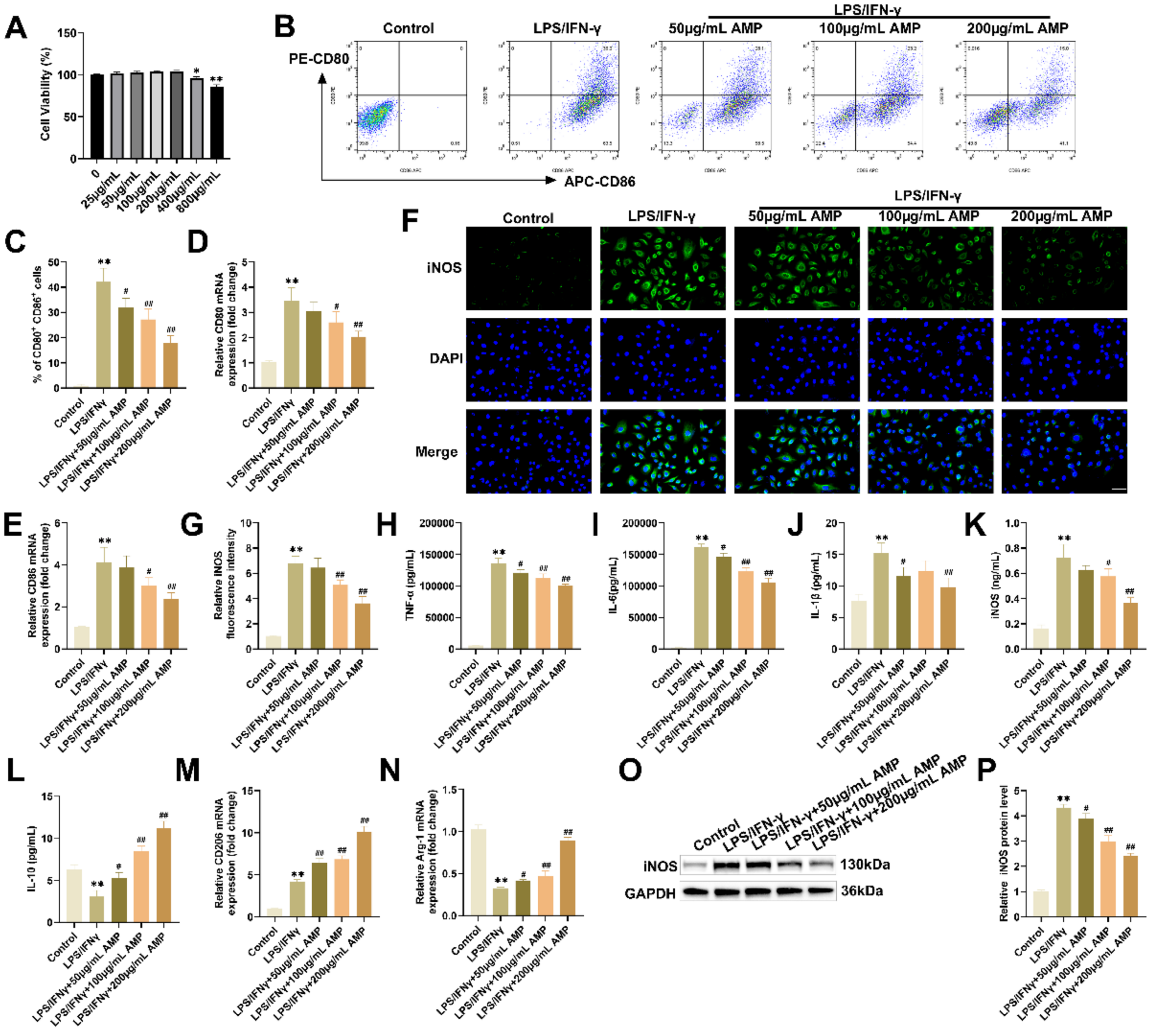
AMP inhibits macrophage M1 polarisation in vitro. (A) MTT assay detecting the viability of RAW264.7 cells at different AMP concentrations. (B, C) Flow cytometry detecting M1 macrophage markers (CD80, CD86) expression in RAW264.7 cells after LPS/IFNγ and AMP treatment, evaluating the effect of different AMP concentrations (50, 100, 200 μg/mL) on LPS/IFNγ‐induced M1 macrophage polarisation. (D, E) qPCR detecting CD80 and CD86 mRNA expression in RAW264.7 cells from each group, assessing the impact of different AMP concentrations on LPS/IFNγ‐induced M1 macrophage polarisation. (F, G) Immunofluorescence detecting M1 macrophage marker (iNOS) expression in each group, evaluating the effect of different AMP concentrations on M1 macrophages. Scale bar 50 μm. (H–L) ELISA quantification of pro‐inflammatory (TNF‐α, IL‐6, IL‐1β, iNOS) and anti‐inflammatory (IL‐10) cytokine levels in LPS/IFNγ‐stimulated RAW264.7 macrophages was performed to evaluate AMP's dose‐dependent immunomodulatory effects. (M, N) qPCR analysis of M2 macrophage markers (IL‐10, CD206, Arg‐1) was performed to assess AMP's dose‐dependent effects on M2 polarisation in LPS/IFNγ‐stimulated RAW264.7 cells. (O, P) Western blot detecting iNOS protein expression in each group, assessing the impact of different AMP concentrations on M1 macrophage polarisation in LPS/IFNγ‐induced RAW264.7 cells. Data are presented as mean ± SD from three biologically independent experiments (*n* = 3). *p < 0.05, **p < 0.01 versus control; ^#^ *p* < 0.05, ^##^ *p* < 0.01 versus LPS/IFNγ.

To determine the optimal AMP concentration range, RAW264.7 cell viability was assessed using MTT assay following treatment with varying AMP doses (25, 50, 100, 200, 400, 800 μg/mL). The data revealed that lower concentrations (25, 50, 100, 200 μg/mL) moderately promoted cellular proliferation, whereas higher doses (400, 800 μg/mL) exhibited marked cytotoxicity, particularly at 800 μg/mL where viability dropped below 90% (Figure 6A). Flow cytometry revealed that in the LPS/IFNγ‐induced RAW264.7 cells in vitro LF model, M1 macrophages (CD80^+^CD86^+^) significantly increased. AMP treatment dose‐dependently reduced M1 macrophages (Figure 6B,C). qPCR, immunofluorescence and Western blot experiments further confirmed that after LPS/IFNγ induction, CD80, CD86 and iNOS expression significantly increased, while AMP treatment dose‐dependently reduced CD80, CD86 and iNOS expression levels (Figure 6D–GO,P). ELISA results indicated that the LPS/IFNγ group showed significantly elevated levels of pro‐inflammatory factors (TNF‐α, IL‐6, IL‐1β, iNOS) and significantly decreased IL‐10 (anti‐inflammatory factor) levels. AMP administration demonstrated concentration‐dependent modulation of inflammatory mediators, suppressing pro‐inflammatory cytokines while elevating anti‐inflammatory factors (Figure 6H–L). qPCR results demonstrated that LPS/IFNγ‐induced macrophages showed significantly reduced M2 macrophage polarisation markers (CD206, Arg‐1), whereas AMP treatment dose‐dependently significantly increased CD206 and Arg‐1 expression levels (Figure 6M,N). These results suggest that AMP can inhibit M1 macrophage polarisation and promote macrophage polarisation towards the M2 phenotype.

### 3.7 AMP Suppresses Macrophage M1 Polarisation and HSCs Activation by Activating the AMPK/Nrf2/HO‐1 Signalling Pathway

**FIGURE 7:**
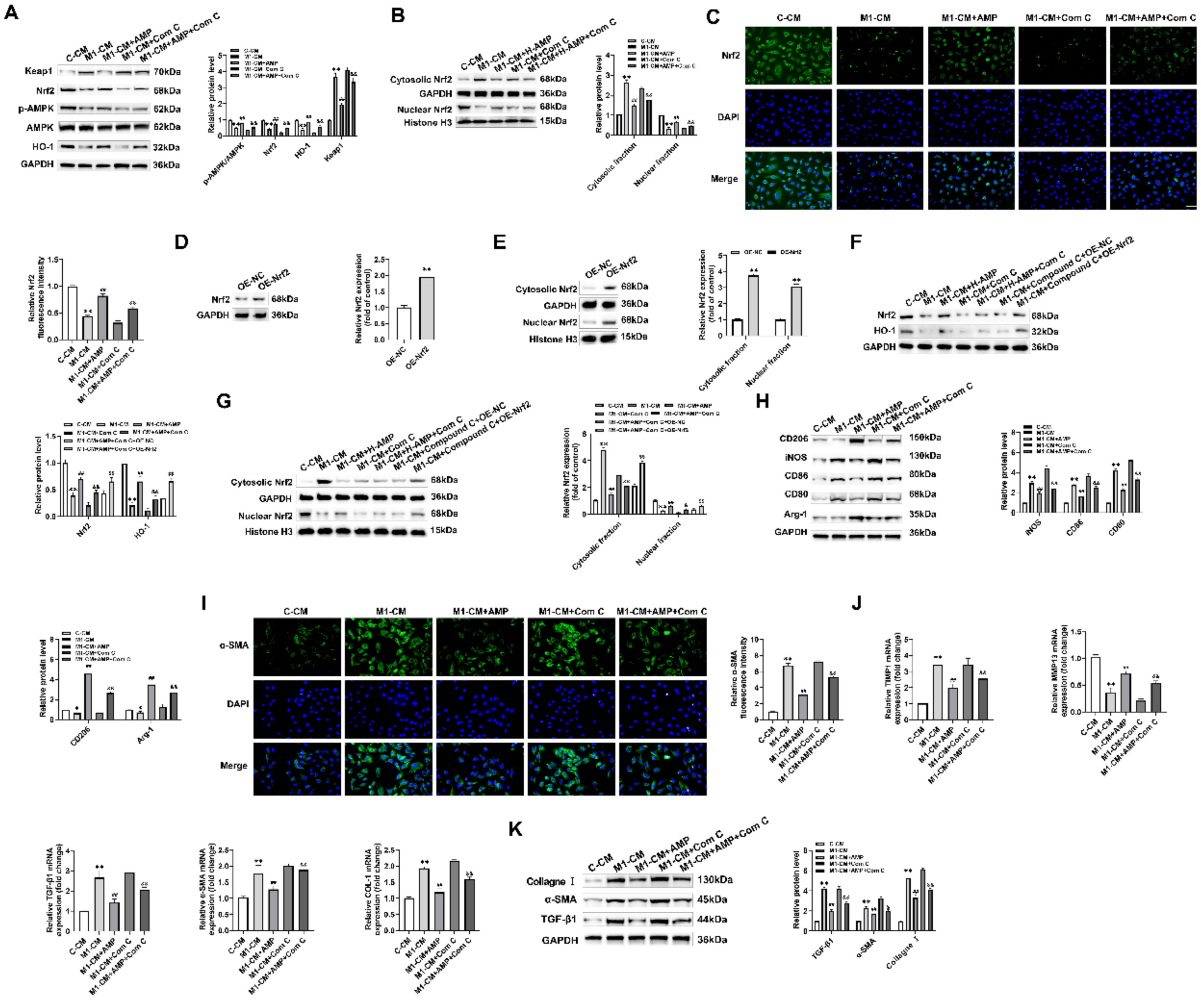
AMP inhibits macrophage M1 polarisation and HSCs activation by activating AMPK/Nrf2/HO‐1 signalling pathway. (A) The expression levels of p‐AMPK, AMPK, Nrf2, Keap1, HO‐1 and other proteins in the AMPK/Nrf2/HO‐1 signalling pathway of RAW264.7 cells in each group were detected by Western blot. (B) Western blot analysis was conducted to determine the levels of Nrf2 protein in the cytoplasm and nucleus of cells from each group. (C) Immunofluorescence detection of Nrf2 expression in RAW264.7 cells. Scale bar 50 μm. (D, E) Through Western blot experiments, the total Nrf2 protein levels in the cells and the expression levels of Nrf2 protein in the cytoplasm and nucleus were detected after transfection with Nrf2 overexpression plasmids. (F, G) Western blot analysis was used to determine the total protein expression of Nrf2 and HO‐1 in IS‐1 cells from each group, as well as the expression levels of Nrf2 protein in the cytoplasm and nucleus. (H) Western blot detecting protein expression of M1 markers (iNOS, CD80, CD86) and M2 markers (CD206, Arg‐1), evaluating AMP's impact on RAW264.7 cell M1 polarisation. (I) Immunofluorescence detection of α‐SMA expression in JS‐1 cells, assessing AMP's effect on JS‐1 cell activation induced by M1 macrophages. Scale bar 50 μm. (J) qPCR analysis of MMP13, TIMP1, TGF‐β1, α‐SMA and Collagen I mRNA expression in JS‐1 cell, evaluating AMP's impact on LF at the RNA level. (K) Western blot detecting protein expression levels of Collagen I, α‐SMA and TGF‐β1, assessing AMP's effect on LF at the protein level. Data are presented as mean ± SD from three biologically independent experiments (*n* = 3). *p < 0.05, **p < 0.01 versus C‐CM; ^##^ *p* < 0.01 versus M1‐CM; ^&&^ *p* < 0.01 versus M1‐CM+AMP.

This investigation employed a LPS/IFNγ‐stimulated co‐culture system of JS‐1 and RAW264.7 cells, combined with pharmacological interventions (AMP, Com C), to elucidate the mechanistic basis of AMP's dual effects on M1 macrophage polarisation and HSCs activation. Western blot analysis demonstrated that LPS/IFNγ‐induced M1 polarisation markedly upregulated key components of the AMPK/Nrf2/HO‐1 pathway (phosphorylated AMPK, Nrf2 and HO‐1), while downregulating the negative regulator Keap1. AMP treatment could reverse these changes, whereas Com C treatment showed no significant alterations. After combined AMP and Com C treatment, compared to the M1‐CM + AMP group, AMP's regulatory effect was significantly weakened (Figure 7A). The results of Western blot and immunofluorescence assays showed that M1‐CM treatment decreased the nuclear level of Nrf2 in RAW264.7 cells and increased the cytoplasmic level of Nrf2; AMP treatment significantly promoted the nuclear translocation of Nrf2; and the combined treatment of Com C and AMP inhibited the nuclear translocation of Nrf2 (Figure 7B,C). To further explore the AMPK/NRF2/HO‐1 signalling pathway, this study constructed an overexpressed Nrf2 plasmid and confirmed that it could effectively upregulate the expression of Nrf2 protein (Figure 4D,E). Overexpression of Nrf2 on the basis of AMP + Com C could significantly upregulate the protein expression of Nrf2 and HO‐1 and restore the nuclear level of Nrf2 (Figure 4F,G). These findings suggest that M1 macrophage polarisation can suppress the AMPK/Nrf2/HO‐1 signalling pathway, while AMP can activate this pathway and Com C attenuates AMP's activation.

Western blot results demonstrated that AMP treatment effectively reversed the M1‐polarised phenotype, showing marked reduction in iNOS, CD80 and CD86 expression coupled with concurrent elevation of CD206 and Arg‐1 levels relative to M1‐CM controls. After Com C treatment, iNOS, CD80 and CD86 protein levels slightly increased compared to the M1‐CM group, while CD206 and Arg‐1 were slightly downregulated. Notably, when AMP and Com C were combined, iNOS, CD80 and CD86 protein levels significantly increased, while CD206 and Arg‐1 protein levels significantly decreased (Figure 7H). The data demonstrate that AMP modulates macrophage polarisation by suppressing M1 phenotype development while enhancing M2 differentiation through AMPK/Nrf2/HO‐1 pathway activation. Immunofluorescence, qPCR and Western blot further analysed that JS‐1 cells induced by macrophage M1 polarisation showed significantly increased expression levels of α‐SMA, TIMP1, TGF‐β1 and Collagen 1, with significantly decreased MMP13 expression, indicating significant LF. After AMP treatment, α‐SMA, TIMP1, TGF‐β1 and Collagen 1 expression levels were significantly reduced, while MMP13 expression significantly increased, demonstrating that AMP significantly suppresses HSCs activation, promotes ECM metabolism balance and improves LF. Compared to M1‐CM, Com C treatment showed no significant changes in α‐SMA, TIMP1, TGF‐β1, Collagen 1 and MMP13 expression levels, but combined treatment weakened AMP's inhibitory effect (Figure 7I–K). The findings demonstrate that AMP attenuates macrophage M1 polarisation through AMPK/Nrf2/HO‐1 pathway activation, resulting in suppressed HSCs activation, decreased ECM accumulation and consequent amelioration of LF.

## 4 Discussion

LF represents the central pathological feature in the progression of various CLDs. This progressive condition is characterised by excessive ECM accumulation resulting from sustained injury‐repair cycles, which disrupts hepatic architecture and compromises liver function [21, 22]. Hepatic fibrogenesis involves coordinated immune cell recruitment, where parenchymal damage initiates sequential infiltration of innate and adaptive immune components including monocyte‐derived macrophages, neutrophils, NKT cells and dendritic cells [1, 23]. These activated immune cells, by releasing reactive oxygen species (ROS), pro‐inflammatory cytokines and key pro‐fibrotic mediators (TGF‐β), activate HSCs to differentiate into MFBs, promote abnormal ECM deposition and ultimately lead to LF and even liver cirrhosis [24]. In cholestatic LF, elevated toxic bile acid levels in the liver and plasma induce hepatocyte and biliary epithelial cell damage and apoptosis. This process not only leads to bile duct proliferation and widespread inflammation but also activates portal fibroblasts (PF) and HSCs, promoting massive Collagen 1 production [25, 26]. Hepatocellular injury and inflammatory responses caused by cholestasis further accelerate LF progression through complex cytokine networks and inflammatory cascades. Current evidence indicates that the Sorafenib‐Fluvastatin combination attenuates hepatic fibrogenesis in rodent models by inhibiting the TGF‐β1/Smad3 pathway, manifested by reduced collagen accumulation and α‐SMA suppression [27], this approach may cause adverse reactions such as diarrhoea and hypertension, limiting its clinical application. Therefore, identifying novel therapeutic targets and developing low‐toxicity, high‐efficacy anti‐LF drugs holds significant scientific and clinical value. In this study, AMP significantly downregulated CCl_4_‐induced mouse liver function damage markers (ALT, AST, ALP) and bile acid metabolism indicators (TBA, TBil), effectively alleviating hepatocellular injury. Simultaneously, AMP significantly suppressed macrophage infiltration, manifested by downregulating F4/80 and CD68 expression and inhibiting pro‐inflammatory factor (IL‐1β, TNF‐α, IL‐6) release while promoting anti‐inflammatory factor (IL‐10) secretion, thereby relieving inflammatory responses and reducing LF severity, specifically reflected in the improvement of key indicators such as HA, PC III, LN and Hyp. Moreover, through histopathological analysis, it was found that AMP can reduce inflammatory cell infiltration, minimise necrotic areas, restore normal hepatocyte morphology and significantly decrease collagen fibre deposition, providing experimental evidence for AMP's potential as an anti‐LF drug.

LF is a dynamic pathological process involving complex collaborative interactions of multiple cell types, with HSCs activation at its core [28]. Under normal physiological states, HSCs maintain a quiescent phenotype characterised by limited proliferative activity and basal collagen production; however, persistent liver injury rapidly induces their transition into an activated, profibrotic phenotype [29]. Activated HSCs extensively express α‐SMA and Collagen 1, secrete ECM components like fibronectin and hyaluronic acid and produce matrix degradation inhibitors, pro‐inflammatory cytokines and chemokines, ultimately leading to pathological ECM deposition and liver tissue remodelling [26, 30]. Once activated, HSCs can maintain their fibrotic state through autocrine signalling. In this process, TGF‐β, a critical mediator produced by infiltrating lymphocytes, monocytes and damaged hepatocytes, triggers an imbalance in ECM deposition and degradation. In the normal liver, multiple macrophage populations derived from the monocyte–macrophage lineage coexist, among which liver‐resident macrophages (Kupffer cells, KCs) highly expressing F4/80 and CD68, demonstrate significant functional characteristics during LF progression [31]. Macrophages can be recruited to injury sites, promoting TGF‐β expression by phagocytosing cell debris and clearing apoptotic cells, thus accelerating HSCs activation and fibrosis progression [32]. For instance, prostate cancer cell‐derived microprotein (PSMP) can promote inflammatory macrophage infiltration, secrete more pro‐inflammatory cytokines and exacerbate LF response [33]. Immunoglobulin‐like Type II receptor α (PILRα) can regulate integrin signal transduction, impede macrophage migration to damaged liver tissue, inhibit hepatic macrophage infiltration, thereby alleviating liver inflammation and mitigating LF [34, 35]. Therefore, inhibiting hepatic macrophage infiltration and recruitment can contribute to LF regression. Experimental evidence demonstrates that AMP exerts anti‐fibrotic effects by upregulating MMP13 to enhance ECM degradation while concurrently suppressing hepatic stellate cell activation (α‐SMA), ECM deposition (Fibronectin, Collagen I), fibrotic progression (TIMP1) and KCs infiltration (F4/80, CD68). Additionally, AMP dose‐dependently attenuates TGF‐β1‐stimulated JS‐1 cell proliferation. Consequently, AMP not only inhibits macrophage infiltration but also suppresses HSCs activation and proliferation.

Macrophages demonstrate remarkable functional plasticity and phenotypic diversity, broadly categorised into M1 and M2 subtypes based on their activation states and biological roles [36]. Macrophages exhibit dual functionality in LF pathogenesis: (1) profibrotic activity through TGF‐β/PDGF‐mediated HSCs activation and MFB differentiation and (2) antifibrotic effects via MMP‐dependent ECM degradation [37, 38, 39]. In this study, AMP treatment downregulated iNOS, CD80 and CD80 expression levels in liver tissues of LF mouse models while upregulating CD206 and Arg‐1 levels. In vitro experiments using LPS/IFN‐γ‐induced macrophage models showed significantly elevated levels of CD80, CD86, TNF‐α, IL‐6, IL‐1β and iNOS, with significantly reduced CD206, Arg‐1 and IL‐10 levels. AMP treatment significantly reversed these changes. The findings demonstrate AMP's ability to suppress pro‐inflammatory M1 polarisation while facilitating anti‐inflammatory M2 phenotypic switching.

During LF development, damage‐associated molecular patterns (DAMPs) released by injured hepatocytes trigger complex immune cascades. Hepatocyte‐derived DAMPs initiate KCs and immune cell activation, sustaining HSCs stimulation through continuous release of inflammatory cytokines and fibrogenic mediators. Simultaneously, this process activates multiple key signal transduction pathways, including TGF‐β1/Smad, MAPK and JAK/STAT, continuously inducing inflammatory responses [40, 41, 42]. Within these complexes signalling networks, the AMP/Nrf2/HO‐1 signalling pathway has garnered significant attention due to its unique regulatory mechanisms in LF research. AMPK, a conserved serine/threonine kinase complex, demonstrates broad cytoprotective effects against oxidative damage and inflammatory responses across multiple biological systems, emerging as a pivotal therapeutic target for metabolic dysfunction‐associated steatotic liver disease (MASLD) and hepatic fibrogenesis [43, 44]. This protective mechanism is closely associated with Nrf2 signal activation. In physiological conditions, cytoplasmic Nrf2 undergoes constitutive Keap1‐mediated degradation, maintaining low basal activity. Under oxidative stress, Nrf2 rapidly translocates to the nucleus, where it activates ARE‐dependent transcription of cytoprotective genes, notably including the critical antioxidant enzyme HO‐1 [45]. HO‐1 catalyses the degradation of heme into CO. Endogenous CO possesses significant anti‐inflammatory characteristics, being capable of inhibiting LPS‐induced pro‐inflammatory cytokine expression while increasing IL‐10 secretion. Conversely, inhibiting Nrf2 activation leads to significantly reduced HO‐1 activity and elevated TGF‐β1 and Smad3 levels, accelerating LF progression [46]. Research has confirmed that kaempferol activates the AMPK/Nrf2 signalling pathway, demonstrating significant antioxidant, anti‐inflammatory and hepatoprotective effects in CCl_4_‐induced liver injury models. The compound suppresses LPS/IFNγ‐induced microglial inflammation via AMPK/Nrf2/HO‐1 activation, attenuating neuroinflammatory mediator release [47]. Hemin can activate AMPK phosphorylation, upregulate HO‐1 expression, inhibit inflammatory responses and HSCs activation and reduce ECM deposition, thus alleviating bleomycin‐induced pulmonary fibrosis [48]. Our integrated in vivo and in vitro findings demonstrate that AMP effectively activates the AMPK/Nrf2/HO‐1 pathway, as evidenced by enhanced AMPK phosphorylation and subsequent upregulation of both Nrf2 and HO‐1 expression. More critically, AMP inhibits pro‐inflammatory factor release, increases anti‐inflammatory factor secretion, downregulates macrophage M1 markers (iNOS, CD80, CD86), upregulates macrophage M2 markers (CD206, Arg‐1), reduces HSCs activation markers (α‐SMA) and decreases ECM marker protein (Fibronectin, Collagen 1) expression. The therapeutic effects of AMP were abolished upon AMPK inhibition, validating its dependence on AMPK/Nrf2/HO‐1 pathway activation. Through modulating macrophage polarisation (suppressing M1 and promoting M2), AMP effectively attenuates HSCs activation and hepatic inflammation, ultimately ameliorating fibrotic progression. Notably, AMP‐mediated pathway activation coincided with Keap1 downregulation, confirming its pivotal role as a negative regulator of Nrf2 signalling. However, this study has certain limitations. First, animal models cannot fully replicate the complex pathological processes of human LF. Future research should incorporate human organoids, primary hepatocytes or clinical liver tissue samples to further validate AMP's antifibrotic effects and enhance its clinical translational potential. Furthermore, although our in vitro experiments demonstrated that AMP can inhibit macrophage M1 polarisation by activating the AMPK/Nrf2/HO‐1 signalling pathway, thereby indirectly improving HSC activation, this study has not yet conducted an in‐depth analysis of the detection specificity and sensitivity of AMP's direct regulation of HSC activation signalling pathways. Future work will involve examining key regulatory molecules upstream and downstream of this pathway, combined with pathway‐specific gene interference techniques, to systematically elucidate AMP's network of action mechanisms. Secondly, this study did not conduct pharmacokinetic research on AMP. Future work should clarify its absorption, distribution, metabolism and excretion characteristics to provide a basis for evaluating its in vivo efficacy and safety. Finally, a comprehensive HPLC/UPLC fingerprint and multi‐component quantitative method have not yet been established. Future research will further improve chemical quality control to promote the clinical translation and broader validation of these findings.

## 5 Conclusion

In summary, AMP effectively inhibits macrophage M1 polarisation and HSCs activation by triggering the AMPK/Nrf2/HO‐1 axis, significantly reducing macrophage infiltration and abnormal ECM deposition, thereby suppressing inflammatory cascade responses and alleviating liver damage and LF progression. This study not only establishes a critical theoretical foundation for AMP therapeutic drug development but also provides important molecular targets for CLD treatment. However, this research has certain limitations, as AMP's regulatory mechanism is not solely dependent on a single signalling pathway. Future studies should delve deeper into exploring its complex regulatory mechanisms within multidimensional networks to comprehensively reveal its potential therapeutic value.

## Author Contributions

**Zukuan Chang:** investigation. **Shixing Li:** writing – original draft, writing – review and editing. **Jinzhan Liu:** formal analysis. **Huilin Lu:** methodology.

## Funding

The authors have nothing to report.

## Ethics Statement

This experiment was approved by Xinxiang Central Hospital Ethics Committee.

## Conflicts of Interest

The authors declare no conflicts of interest.

## Data Availability

The data that support the findings of this study are available from the corresponding author upon reasonable request.
